# Adrenocortical carcinoma secreting cortisol, androgens and aldosterone: a case report

**DOI:** 10.4076/1757-1626-2-8951

**Published:** 2009-09-10

**Authors:** Melpomeni Peppa, Vasilios Pikounis, Georgios Papaxoinis, Anastasios Macheras, Theofanis Economopoulos, Sotirios A Raptis, Dimitrios Hadjidakis

**Affiliations:** 1 Endocrine Unit, Second Department of Internal Medicine-Propaedeutic, Research Institute and Diabetes Center, Athens University Medical School, 'Attikon' University Hospital, 1 Rimini Str, Athens, 12462, Greece; 2 Third Surgery Department, Athens University Medical School, 'Attikon' University Hospital, 1 Rimini Str, Athens, 12462, Greece; 3 Hellenic National Diabetes Center for Research, Prevention and Treatment of Diabetes and its Complications, H.N.D.C, 3 Ploutarchou Str, Athens, 10675, Greece

## Abstract

**Introduction:**

Adrenocortical carcinoma is a rare malignancy with a poor prognosis and presents with mass effects and less often with signs of hormone excess (approximately 60% of all adrenocortical carcinoma's). Hormonally active adrenocortical carcinomas most commonly secrete cortisol while the co-secretion of multiple steroid hormones is rare.

**Case presentation:**

We report the case of a 59 year-old woman with a medical history of hyperaldosteronism due to a right adrenal adenoma. During follow up, she showed symptoms of hypercortisolism and hyperandrogenemia and a rapid growth of the adrenal mass. She underwent right adrenalectomy and the histology revealed the presence of an adrenocortical carcinoma. Six months post-operatively being on treatment with mitotane, she was diagnosed of metastatic disease to the liver. She underwent right hepatectomy and was started on systemic chemotherapy, with no signs of tumour recurrence during the following six months.

**Conclusion:**

The hormonal status should be carefully investigated in all cases of suspected adrenocortical carcinoma, as the pattern of hormone secretion may be a clue to the malignancy of an adrenal lesion. In addition, more data are needed to clarify the clinical and prognostic significance of the combined production of all adrenocortical hormones affecting either the survival or the quality of life of adrenocortical carcinoma patients.

## Introduction

Adrenocortical carcinoma (ACC) represents a rare malignancy accounting for 0.05-0.2% of all cancers with a poor prognosis depending on the stage of the disease and the completeness of the resection [1,2]. Approximately 60% of ACC's are hormone-secreting and the steroid profile often displays a wide variety of steroids in ACCs, which may be used as tumour markers to detect metastastic disease. Most commonly ACC secretes cortisol while the combined secretion of all adrenocortical hormones is quite rare with unknown either clinical or prognostic significance [3]-[5].

## Case presentation

In September 2006, a 59-year old Greek Caucasian woman presented to her local hospital due to a hypertensive crisis (blood pressure: 170/100 mmHg). Endocrinological investigation, revealed marked hypokalemia (serum potassium: 2.7 mEq/l), suppressed renin levels, increased aldosterone levels with an aldosterone/renin ratio >30, findings suggestive of primary hyperaldosteronism. However, cortisol, catecholamines or other hormones were not measured, despite the fact that she was a hypertensive patient. Abdominal CT scan revealed the presence of a solid enlargement of the right adrenal (max diam. 2 cm) with radiological characteristics of benign adenoma while attenuation value and wash-out procedure were not performed, making the diagnosis of "benign adenoma" somewhat arbitrary. The patient was started on spironolactone treatment, with gradual improvement of her blood pressure and serum potassium levels and was suggested of surgical excision of the adrenal adenoma, which she denied.

**Figure 1:**
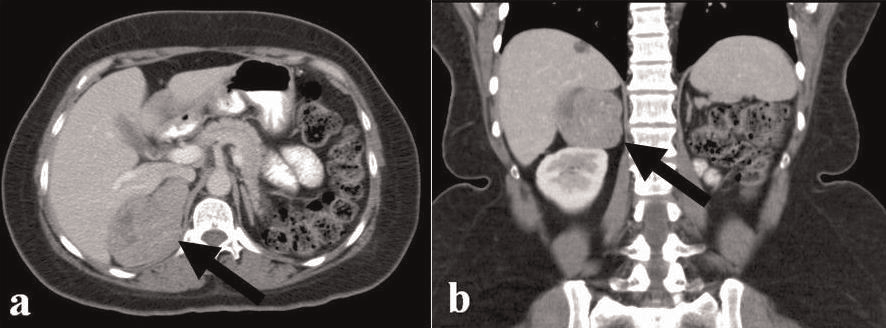
**Abdominal CT scan showing a large right adrenal mass**.(max diam 6.5 cm).

**Figure 2:**
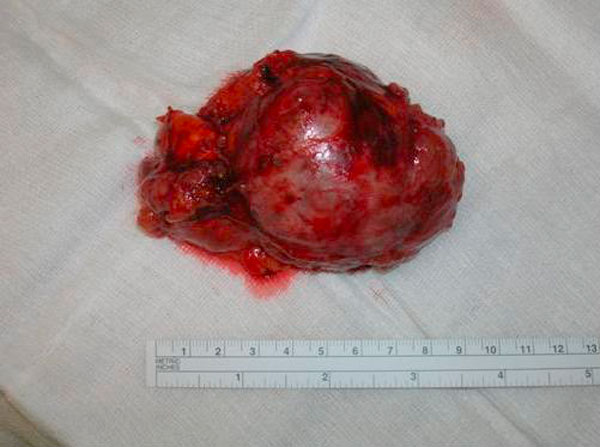
**Macroscopic findings of the surgically removed tumour**.

One-year later, she was referred to our department with signs of sustained hypertension despite spironolactone treatment, mild hirsutism, irregular vaginal bleeding, increased body weight and central adiposity. Abdominal CT and MRI scan showed a large right adrenal mass (6.1 × 6.5 × 5.5 cm), with morphological characteristics suggestive of carcinoma (increased attenuation value of the lesion, low relative enhancement washout) (Figure 1a,b). Hormonal evaluation showed increased 24-hour urinary free cortisol excretion, loss of circardian rhythm of cortisol, cortisol non-suppression in dexamethasone test and undetectable ACTH levels, suggestive of hypercortisolism of adrenal origin. In addition, hyperandrogenemia was found, reflected in increased androgen levels, in particular total and free testosterone, Δ4 androstenedione and 17 OH progesterone. (Table 1) Despite being on spironolactone treatment, the patient had increased aldosterone levels (17.8 ng/dl, normal 0.8-13), while renin was within normal values (3.6, normal 1.5-5.7 ng/ml/h). She underwent right adrenalectomy and histology confirmed the diagnosis of ACC (Figure 2) with a Ki-67 proliferation index of approximately 25%. The patient was subsequently started on mitotane treatment. Despite the fact that scintigraphy was not performed postoperative normalization of the hormonal profile was indicative of the multi-hormonal secretion by the ACC (Table 1).

**Table 1:** Hormonal profile of the patient with ACC before and after surgical treatment

Hormonal profile	Before	After	Normal range
Plasma renin activity (PRA) (ng/ml/h)	0.1	5.3	1.5-5.7
Aldosterone (ng/dl)	39	12.9	0.8-13
Aldosterone/Renin ratio	390	2.4	
Basal serum cortisol 8 am-12 pm (ug/dl)	13.5-12	12	6.2-19.4
Urinary free cortisol (ug/24hrs)	146	65	20-130
Plasma ACTH (pg/ml)	<1	24.5	5.0-60
Serum testosterone (ng/dl)	229	5.91	6.0-82
Serum free testosterone (pg/ml)	0.4	0.3	0.3
Serum Δ4-androstendione (ng/ml)	7.7	2.3	0.3-3.3
Serum 17-OH progesterone (ng/ml)	3.4	0.6	0.2-3.3
Serum DHEAs (ug/dl)	61	40	35-430

**Figure 3:**
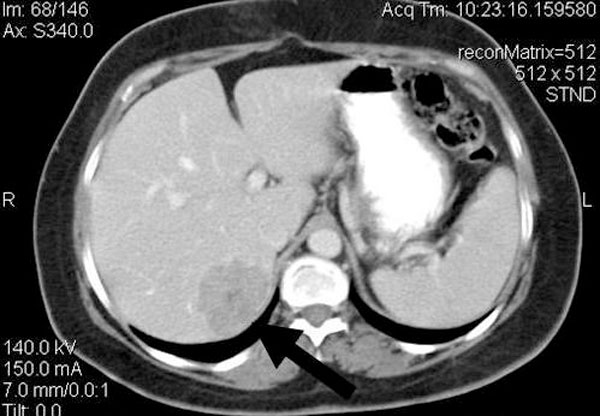
**Abdominal CT scan showing a metastatic lesion in the right hepatic lobe**. (max diam 2.6 cm).

Six months after surgery, she showed signs of hypertension recurrence accompanied by hypokalemia (serum potassium: 2.9 mEq/l) and hypernatremia (serum sodium: 150 mEq/l). Abdominal CT scan revealed a metastatic lesion in the right hepatic lobe (diam 2.6 cm) (Figure 3). The patient was referred to an oncology hospital, where she underwent right hepatectomy and was started on systemic chemotherapy, with no signs of tumour recurrence in the following six months according to the hospital's medical report. The Pathology Department which analyzed the hepatic tumor histology confirmed that it was an ACC metastasis. However, data regarding the exact histological findings of the hepatic metastasis in detail, are not available.

## Discussion

We report a patient with ACC and abnormal secretion of all adrenocortical hormones, namely glucocorticoids, mineralocorticoids and androgens. Although she was initially presented with hyperaldosteronism and radiological characteristics suggesting a benign adenoma, during a rather short-time follow up, she showed hypersecretion of all adrenal hormones, in parallel with an aggressive behavior of the ACC manifested by the rapid growth of the adrenal tumour and hepatic metastasis.

In our case, only aldosterone and renin levels were measured during her initial evaluation, despite the fact that other hormones such as cortisol and catecholamines should be measured in a hypertensive patient. However, quantitative and/or qualitative changes in hormone secretion by an adrenal tumour are not uncommon [6]. It has been reported that ACC cases produce hormones and the steroid profile often displays a wide variety of steroids in ACCs, which may be used as tumour markers to detect metastastic disease. Most commonly ACC produces cortisol (50%), followed by androgens while aldosterone production is quite rare (<2%) [4]. Less frequently, ACC produces combined hormonal syndromes most commonly cortisol with androgens (up to 46.7% of cases) while the combined hypersecretion of all adrenocortical hormones occurs rarely [3]-[5]. Moreover, increased cortisol production has been characterized as a poor prognostic factor for ACC and the overall prognosis seems to be improved with o,p'DDD treatment [7] However, there are not any certain clues regarding the natural course and the clinical significance of increased co-secretion of all steroid hormones in ACC cases [3]-[5].

In addition, the temporal development from a 2 cm to a 6 cm tumour being malignant has rarely been reported. Most studies of incidentalomas have a portion of patients found to suffer from ACC, but their tumours are large (at least >4 cm) or grow rapidly (5-15 cm in 3 months) but not one which seem to have a quite modest growth rate (3-fold) in 1 year, as the present case. Our case in support of other cases and studies indicate that the growth rate of an ACC is largely unknown and varies widely between different patients due mostly to the fact that they are treated when they are found and not followed as the present case.

## Conclusion

In conclusion, the hormonal status should be carefully investigated in all cases of suspected ACC, as the pattern of hormone secretion may be a clue to the malignancy of an adrenal lesion [8]. In addition, more data are needed to clarify the growth rate of the ACC and the clinical and prognostic significance of the combined production of all adrenocortical hormones affecting either the survival or the quality of life of ACC patients.

## Abbreviations

ACC: adrenocortical carcinoma; ACTH: adrenocorticotropic hormone; CT: computed tomography; DHEAs: dehydroepiandrosterone sulfate; MRI: magnetic resonance imaging; o,p'DDD: ortho, para', dichloro-, diphenyl-, dichloro ethane or mitotane.

## Consent

Written informed consent was obtained from the patient for publication of this case report and any accompanying images. A copy of the written consent is available for review by the journal's Editor-in-Chief.

## Competing interests

The authors declare that they have no competing interests.

## Authors' contributions

MP conceived the study, collected data and drafted the manuscript. VP and GP helped with the patient care and the literature reviewing. AM carried out the operation. TE, SAR and DJ critically revised the paper. All authors read and approved the final version of the manuscript.
